# Predicting the presence of 4th ventricular outlet obstruction in Chiari I Malformation

**DOI:** 10.1007/s00381-024-06482-w

**Published:** 2024-06-07

**Authors:** Kenneth S. Paik, Caroline Caudill, Anastasia Arynchyna-Smith, Brandon G. Rocque, Curtis J. Rozzelle

**Affiliations:** 1 Division of Pediatric Neurosurgery, Department of Neurosurgery, University of Alabama at Birmingham, Birmingham, AL USA; 2 Marnix E. Heersink School of Medicine, University of Alabama at Birmingham, Birmingham, AL USA

**Keywords:** Chiari decompression, Syringomyelia, Arachnoid veil, Obstruction

## Abstract

**Introduction:**

A subset of children with Chiari 1 malformation (CM-1) have a 4th ventricle arachnoid veil—a thin membrane covering the outlet of the 4th ventricle. Studies suggest that failure to disrupt this veil during posterior fossa decompression can reduce the likelihood of syringomyelia resolution. However, there is no reliable method for predicting the presence of the veil without direct surgical exploration. This study aims to evaluate the association between pre-operative symptoms, radiographic measurements, and the arachnoid veil.

**Methods:**

A retrospective review of an institutional database of children evaluated for CM-I was conducted. For patients treated with surgery, operative notes were reviewed to determine if an arachnoid veil was present. Logistic regression was used to test for relationship of clinical variables and radiographic measurements with the presence of an arachnoid veil.

**Results:**

Out of 997 children with CM-1, 226 surgical patients were included in the analysis after excluding those with inadequate documentation. An arachnoid veil was found in 23 patients (10.2%). Larger syrinx, spinal canal, and thecal sac diameters were significantly associated with the presence of a veil, with odds ratios of 1.23 (95% CI 1.2–1.48; p = 0.03), 1.27 (95% CI 1.02–1.59; p = 0.03), and 1.35 (95% CI 1.03–1.77; p = 0.03), respectively. No significant associations were found with any signs or symptoms.

**Conclusions:**

Arachnoid veil was present in 10% of cases. Radiographic measurements indicating larger syrinx size were the only variables found to be significantly associated with an arachnoid veil. Exploration of the 4th ventricular outlet is recommended for CM-I decompression in the setting of expansile syringomyelia.

## Introduction

CM-1 is commonly defined as cerebellar tonsillar position outside the foramen magnum [1]. An arachnoid veil is a thin glial membrane that covers the midline outlet foramen of the fourth ventricle (foramen of Magendie) [2, 3]. While the significance of an arachnoid veil is not entirely clear, it has been suggested that the presence of an arachnoid veil disrupts CSF flow across the fourth ventricle outlet. This, in turn, could be a risk factor for development of syringomyelia [2]. The exact relationship between arachnoid veil, CM-1, and syringomyelia is not fully understood. Because surgical inspection is required to identify the presence of an arachnoid veil, it is possible that not all people with CM-1 and arachnoid veil will develop syringomyelia. However, when retroactively evaluating surgical cases in which an arachnoid veil was reported, a preoperative syrinx was often present [2].

CM-1 can be treated surgically or managed conservatively depending on the severity of symptoms and the extent of the malformation. Bone-only decompression surgery and decompression with duraplasty are both considered acceptable treatments, effective in most cases [4, 5]. However, bone-only decompression surgery does not permit direct inspection for the presence of an arachnoid veil. If a patient with an arachnoid veil undergoes bone-only decompression, the procedure may fail to relieve their symptoms or achieve syrinx reduction/resolution, necessitating a second surgery. Dural opening (followed by duraplasty) enables visual examination of the fourth ventricular outlet and identification of the veil, which can then be opened to facilitate unrestricted cerebral spinal fluid (CSF) flow, thereby alleviating symptoms and/or syringomyelia [6].

The aim of this study is to investigate the association between pre-operative symptoms and radiographic measurements with the presence of a fourth ventricular outlet arachnoid veil. If such association exists, it might allow surgeons to plan for intradural exploration in cases where a veil is more likely, reducing the potential need for repeat surgery in some cases.

## Methods

Ethical approval was provided by the institutional review board at the University of Alabama at Birmingham. n institutional database of all children evaluated for CM-1 by the neurosurgery group at Children’s of Alabama was used to identify subjects for this study. The details of the creation of this cohort of patients are available in a previous publication [7]. Patients who were evaluated by a neurosurgeon at least once during this time at Children’s of Alabama in Birmingham, Alabama were selected for retrospective chart review.

Data collection was performed by neurosurgery residents or graduate students who were trained by neurosurgeons on how to evaluate medical records and radiographic imaging. If there was uncertainty during chart review or radiographic measurement, an attending neurosurgeon was consulted to provide the final decision.

Because presence or absence of an arachnoid veil cannot be determined without surgical intervention, patients in this original list who did not undergo a Chiari decompression were excluded. All patients initially screened and analyzed in this study underwent posterior fossa decompression with duraplasty.

To determine the presence of an arachnoid veil, operative notes dictated by the neurosurgeon during the Chiari decompression were examined. In the event where patients experienced multiple intradural procedures, the operative notes dictated during the initial Chiari decompression were selected for review and data collection. If these operative notes did not mention opening the dura and searching for an arachnoid veil (or any language related to a veil such as “membrane”, “adhesion”, “web”) or exploring the outlet of the fourth ventricle, the patient was considered to have insufficient documentation regarding presence or absence of a veil and was excluded. Therefore, all included subjects had explicit documentation in the operative note of whether an arachnoid veil was present or absent.

### Classification of clinical and radiographic variables

In addition to determining the presence or absence of an arachnoid veil, chart review was used to identify presenting clinical signs and symptoms including whether the patient reported tussive headaches (headaches specifically caused by cough, sneeze, Valsalva, or similar maneuvers), non-tussive headaches, paresthesia, lower cranial nerve dysfunction (dysphagia, dysarthria, sensory disturbances), or sleep study abnormalities (central or obstructive sleep apnea).

To identify radiographic variables, the most-recent MRI study before surgery was reviewed using Syngo Plaza diagnostic imaging software (Siemens Healthcare, USA). Acceptable imaging types included MRI sagittal T1 or T2 slices and could be cervical or full spine images. Imaging variables were measured using the standard definitions and measurements according to the National Institute of Neurological Disorders and Stroke Common Data Elements (NINDS) for Chiari. Variables included tonsillar position, syrinx position, length, and diameter (if a syrinx was present), diameter of spinal cord, diameter of thecal sac, clivo-axial angle (CXA) degrees, posterior basion-C2 distance (pBC2), classification of a Chiari 1.5, clivus length, supraocciput length, foramen magnum diameter, superior posterior fossa length, tentorial angle, and basilar invagination [8].

### Statistical analysis

Missing data was purposefully coded as such to prevent analysis based on incomplete information. Variables with two distinct categories (such as presence of a veil) were coded dichotomously. All statistical analysis were performed using IBM SPSS version 29 software (SPSS Inc., Chicago, IL). Descriptive statistics were used to summarize the cohort. Comparisons were made based on presence or absence of an arachnoid veil.

Univariable logistic regression was performed for each individual dichotomous variable with the presence versus absence of arachnoid veil as the outcome variable. Significance for all tests was based on a p -value less than 0.05.

## Results

**Fig. 1:**
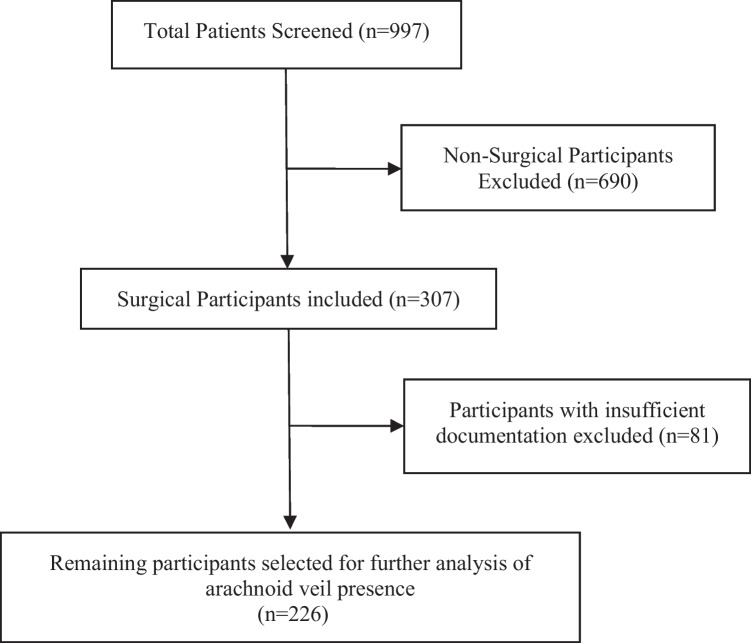
Flow diagram of exclusion and inclusion criteria for participants with and without arachnoid veil

Cohort included 226 pediatric patients after excluding non-surgical patients (n = 690) and patients with inadequate documentation regarding the presence or absence of an arachnoid veil (n = 81) (Fig. 1). Of all patients screened, 123 (54%) were female and 103 (46%) were male. Table 1 provides the details of the cohort composition.

**Table 1:** Descriptive data of the total cohort

	*N* (%)			Univariate Logistic Regression	
	Total Cohort (*N* = 226)	Veil Present (*N* = 23)	Veil Absent (*N* = 203)	Odds Ratio (± 95% CI)	*p*
Gender					
Female	123(54%)	15(65%)	108(53%)	0.61 (0.25–1.49)	0.23
Male	103(46%)	8(35%)	95(47%)	*ref*	
Race					
White	161(71%)	15(65%)	146(72%)	0.94 (0.70–1.26)	0.66
Black	34(15%)	4(17%)	30(15%)	*ref*	
Other or Unknown	31(14%)	4(17%)	27(13%)	*ref*	
Insurance					
Public	47(21%)	2(9%)	45(22%)	2.92 (0.65–13.12)	0.16
Private	148(65%)	17(74%)	131(65%)	*ref*	
Other or Unknown	31(14%)	4(17%)	27(13%)	*ref*	
Age at Surgery	10(5)	12(4)	10(5)	1.06 (0.97–1.16)	0.21

**Fig. 2:**
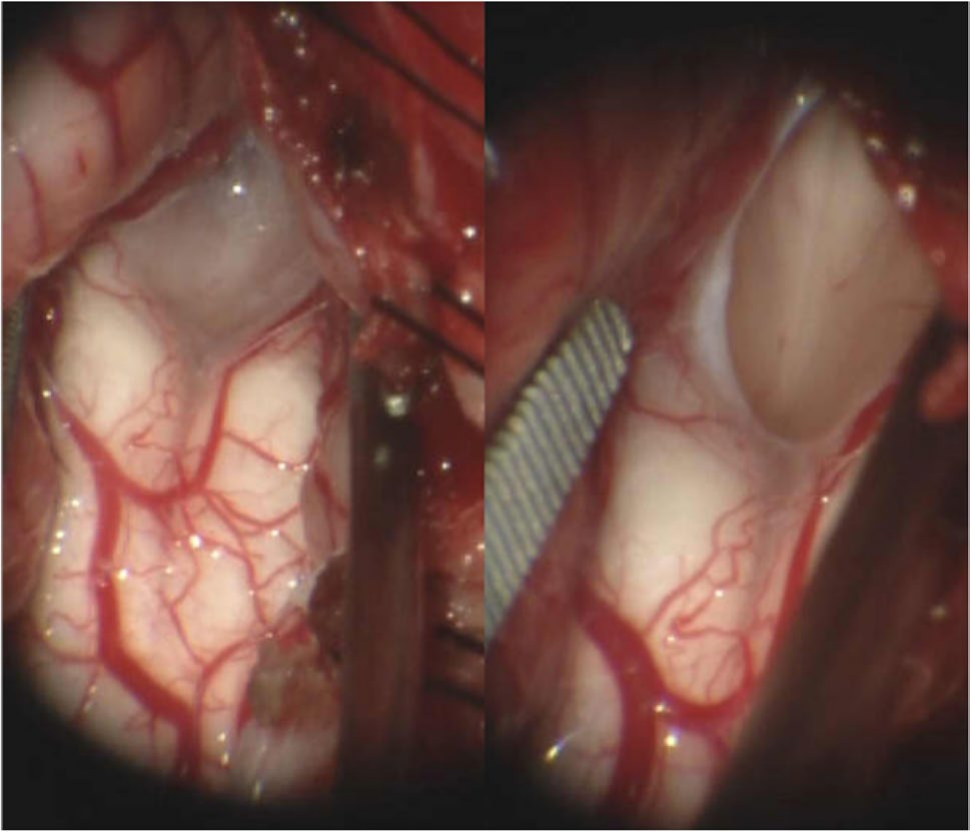
Intradural exposure demonstrating an arachnoid veil causing fourth ventricular outlet obstruction (left). Opening of the veil and relieving the obstruction (right)

An arachnoid veil was present in 23 patients (10%). Of cases where a veil was noted, 20 out of 23 had pre-operative syringomyelia (87%, OR 2.67 95% 0.76–9.32; p = 0.12) (Table 2). Univariate logistic regression did not identify any clinical variables that showed significant association with presence of an arachnoid veil (Table 3). Among radiographic measurements, only those describing the size and extent of spinal syrinx were significant. These included larger maximum transverse diameter of the syrinx (OR 1.23, 95% 1.2–1.48; p = 0.03), transverse diameter of the spinal canal (OR 1.27, 95% 1.02–1.59; p = 0.03), and transverse diameter of the thecal sac (OR 1.35, 95% 1.03–1.77; p = 0.03). Additionally, a presence of a holocord syrinx (defined as extending through the entire spinal cord, from cervical to near the conus medullaris) showed significant association with veil presence (OR 2.70, 95% 1.09–6.70; p = 0.03). Other radiographic measurements detailed in Table 3 did not demonstrate a statistically significant association with the presence of the veil (Fig. 2).

**Table 2:** Pre-operative symptoms and syrinx description of total cohort [7]

	*N* (%)			Univariate Logistic Regression	
	Total Cohort (*N* = 226)	Veil Present (*N* = 23)	Veil Absent (*N* = 203)	Odds Ratio (± 95% CI)	*p*
Tussive Headache	59(26%)	5(22%)	54(26%)	0.77 (0.27–2.17)	0.62
Non-Tussive Headache or Neck Pain	84(37%)	12(52%)	72(35%)	1.88 (0.81–4.3)	0.14
Lower Cranial Nerve Dysfunction	21(9%)	0(0%)	21(10%)	*null*	
Sleep Study Abnormality	15(7%)	1(4%)	14(7%)	0.61 (0.08–4.89)	0.65
Paresthesia	25(11%)	4(17%)	21(10%)	1.83 (0.57–5.87)	0.31
Syrinx Presence					
Yes	165(73%)	20(87%)	145(71%)	2.67 (0.76–9.32)	0.12
No	61(27%)	3(13%)	58(29%)	*ref*	
Syrinx Position					
Cervical	43(19%)	5(22%)	38(19%)	1.21 (0.42–3.45)	0.73
Thoracic	15(7%)	1(4%)	14(7%)	0.61 (0.08–4.89)	0.65
Holocord	**48(21%)**	**9(39%)**	**39(19%)**	**2.70 (1.09–6.70)**	**0.03**
Cervicothoracic	54(24%)	5(22%)	49(24%)	0.87 (0.31–2.47)	0.80
Prominent Central	5(2%)	0(0%)	5(2%)	*null*	

^**^All measurements are reported in millimeters

**Table 3:** Pre-operative radiographic measurements and surgical descriptors of total cohort [7]

	*N* (% of Sample)			Univariate Logistic Regression	
	Total Cohort (*N* = 226)	Veil Present (*N* = 23)	Veil Absent (*N* = 203)	Odds Ratio (± 95% CI)	*p*
Repeat Decompression	16(7%)	1(4%)	15(7%)	0.57 (0.07–4.52)	0.60
Chiari 1.5	71(31%)	9(39%)	62(31%)	1.98 (0.67–5.89)	0.22
Tonsil Shape					
Round	135(60%)	14(61%)	121(60%)	0.84 (0.31–2.27)	0.73
Pegged	8(3%)	0(0%)	8(4%)	*null*	
Intermediate	16(7%)	1(4%)	15(7%)	1.47 (0.17–12.67)	0.73
	Mean (SD)				
Lowest Tonsillar Position	15(7)	13(5)	15(7)	0.98 (0.9–1.06)	0.54
Maximum Syrinx Diameter	**7(3)**	**9(3)**	**7(4)**	**1.23 (1.2–1.48)**	**0.03**
Length of Syrinx (levels)	11(6)	14(6)	11(6)	1.11 (0.99–1.24)	0.09
Maximum Spinal Cord Diameter	**10(3)**	**12(3)**	**10(3)**	**1.27 (1.02–1.59)**	**0.03**
Maximum Thecal Sac Diameter	**13(2)**	**15(2)**	**13(2)**	**1.35 (1.03–1.77)**	**0.03**
CXA Degrees (Soft Tissue)	135(12)	135(16)	135(12)	1.01 (0.96–1.05)	0.82
pBC2	7(2)	7(2)	7(2)	1.1 (0.85–1.43)	0.46
Clivus	34(8)	32(9)	34(4)	0.97 (0.91–1.03)	0.33
Supraocciput	43(40)	41(5)	44(42)	1 (0.98–1.02)	0.86
Foramen Magnum Diameter	35(4)	36(3)	35(4)	1.02 (0.95–1.09)	0.40
Superior Posterior Fossa Length	83(10)	84(6)	83(11)	1.02 (0.95–1.09)	0.64
Tentorial Angle	89(66)	84(12)	89(67)	1 (0.98–1.02)	0.81
Basilar Invagination	5(3)	5(2)	5(3)	0.86 (0.69–1.07)	0.17

^**^All measurements are reported in millimeters

## Discussion

In the first study to describe the presence of arachnoid veils, it was noted that the veil was not observable on pre-operative imaging [2]. The purpose of this study was to determine if any factors available to the surgeon before surgery were associated with the presence of an arachnoid veil. Foreknowledge of the presence of an arachnoid veil is important, as previous work has shown that failure to disrupt an arachnoid veil, when present, can lead to failure of syrinx resolution. We hypothesized that indirect evidence might exist if associations could be identified between specific preoperative clinical information and radiographic findings that could suggest the presence of an arachnoid veil.

The surgical management of CM-1 has been a topic of debate among neurosurgeons. Central to this debate is whether or not to perform dural opening during surgery. Those in favor of duraplasty argue that it is important to create additional space for the cerebellum and brainstem and to allow for proper evaluation of the presence of veils. However, this approach is associated with higher risk of infection, CSF leakage, and postoperative complications [9–11]. On the other hand, bone-only decompression poses fewer immediate surgical risks, but carries higher recurrence of symptoms, and a greater likelihood of syrinx persistence/progression [12–14]. Importantly, bone-only decompression does not allow for intradural exploration, and therefore misses the opportunity to assess for the presence of an arachnoid veil [2].

Currently, the literature surrounding the arachnoid veil in children with CM-1 is limited. Determining its significance in patients who have not undergone duraplasty presents a challenge. Our analysis reveals positive correlations with imaging findings related to the diameter and extent of the syrinx (holocord syrinx, larger syrinx diameter, larger diameter of the spinal cord). However, no other variables showed association with veil presence. It is important to note that a wider syrinx diameter alone should not be considered as the only determining factor; it may simply be associated with the presence of a veil, or more plausibly, the veil itself could be the primary cause of a larger syrinx. It could also be hypothesized that individuals with a greater spinal canal diameter might manifest a larger syrinx diameter, attributed to prolonged exposure to an expansive syrinx leading to bony remodeling.

## Limitations

Roughly, 25% or 81 patients were described as having insufficient data as described in the operative notes. Analyzing each patient chart, if the operative note did not discuss the presence or absence of the arachnoid veil, they were excluded from this study, and only patient charts that described the veil as being present or absent were included. While we have identified significant association with syrinx size and veil presence, we are not able to identify a clinically useful cutoff value.

## Conclusion

The determination of the presence of arachnoid veils in patients with CM-1 based on clinical features or radiographic findings only is not feasible. However, our study demonstrates a strong link between the size of the syrinx cavity and spinal canal and the presence of arachnoid veils. These findings suggest that, for patients with larger syringes, intradural exploration that allows observation of the fourth ventricle outlet might be indicated to ensure unhindered outflow of CSF. As our understanding of this condition develops, more precise clinical management strategies can be established to improve patient outcomes. In conclusion, further research is needed to elucidate the mechanisms of formation of arachnoid veils and their association with CM-1.

## Data Availability

No datasets were generated or analysed during the current study.
